# Serum protein acidic and rich in cysteine (SPARC) as a prognostic marker in soft tissue sarcomas

**DOI:** 10.1186/2045-3329-4-2

**Published:** 2014-01-31

**Authors:** Sherif S Morgan, Raymond B Nagle, Lee D Cranmer

**Affiliations:** 1 The University of Arizona Cancer Center, 1515 N. Campbell Avenue, Tucson, AZ, USA; 2 Department of Pathology, University of Arizona, Tucson, AZ, USA

**Keywords:** Soft-tissue sarcoma, SPARC, Nanoparticle albumin-encapsulated (NAB)-paclitaxel

## Abstract

**Background:**

Serum protein acidic and rich in cysteine (SPARC) is a matricellular secreted glycoprotein that performs several cellular functions and has been implicated in tumorigenesis in a variety of tumor types. The chemotherapeutic agent nanoparticle albumin-encapsulated (NAB)-paclitaxel has been postulated to exploit SPARC expression to target neoplastic cells. SPARC’s role, and potentially the role of NAB-paclitaxel, in the highly heterogeneous class of soft-tissue sarcomas (STS) has not been investigated. Our objective was to explore the pattern of SPARC expression and its prognostic significance in STS.

**Methods:**

27 tissue specimens representing various STS histologies were stained for SPARC expression by immunohistochemistry (IHC). Staining intensity was scored blindly. Survival was determined from patients’ medical records and analyzed using Kaplan-Meier and log-rank with respect to SPARC expression level.

**Results:**

Elevated SPARC expression was observed in 15/27 (56%) specimens. Overall patient survival segregated strongly based on levels of SPARC expression. Patients who expressed low-to-moderate levels of SPARC exhibited median survival of 22.1 months, while the median survival of patients with moderate-to-high expression levels was 4.4 months (log rank; *p* = 0.0016).

**Conclusions:**

SPARC expression is elevated in a significant proportion of STS specimens analyzed in this study, but it does not appear to correlate with specific STS histologies. Given our limited sample size, we cannot draw definitive conclusions regarding association of SPARC with STS subtype. Overall survival segregates strongly by degree of SPARC expression, with elevated expression being adverse. If validated in a larger study, our results suggest that trials in STS with agents potentially targeting SPARC, such as NAB-paclitaxel, should be stratified by SPARC expression level.

## Background

Soft-tissue sarcomas (STS) are a relatively rare, heterogeneous group of malignancies. In the United States, an estimated 11,410 new cases and 4,390 deaths were anticipated in 2013 from STS [1]. Currently, the most effective treatment for localized STS is surgical resection, which is sometimes combined with adjuvant radiation therapy to improve local control [2]. Both radiation therapy and systemic cytotoxic therapy are used in the treatment of primary disease for adjuvant, neo-adjuvant, and palliative treatments.

Unresectable or metastatic disease occurs in approximately 40-60% of patients [3,4] and generally portends poor prognosis. The median survival of patients with advanced disease is approximately 12 months [5,6]. Treatment of advanced disease relies primarily on a limited repertoire of systemic agents. Doxorubicin, in use since the 1970s, remains the backbone of most sarcoma systemic therapy, but its use is complicated by cardiotoxicity. Further, only a minority of doxorubicin-treated patients (estimated at 26% in a meta-analysis) demonstrates objective responses to treatment [6]. Other cytotoxics frequently employed in sarcoma therapy include ifosfamide, dacarbazine, gemcitabine, and taxanes [7]. With the exception of gastrointestinal stromal tumors (GIST), targeted therapies have played a minor role in the management of STS so far (reviewed in [7]). Treatments available for STS, particularly when disease is unresectable or metastatic, are inadequate and rarely yield durable responses. Virtually all patients will require salvage therapy.

Progress in the treatment of STS has been hampered by its heterogeneity, relative rarity, and our lack of understanding of the underlying biology of STS subtypes. Sarcoma is comprised of at least 50 distinct histological entities [4,8]. The majority of sarcoma cases exhibit multiple complex karyotypic aberrations without consistent patterns within each subtype [4,9]. Even in sarcoma cases that are characterized by specific genetic alterations, using targeted agents have not always led to clinical benefit despite the presence and overexpression of the molecular target [10-13]. Thus, fully understanding the underlying biology and characterizing the mechanisms essential for progression of each STS subtype will likely prove critical to improve the treatment options for this group of diseases.

Serum protein acidic and rich in cysteine (SPARC), also known as osteonectin or basement-membrane-40 (BM-40), is a matricellular secreted glycoprotein. Initially, SPARC was identified as a bone-specific phospho-protein [14], but later was identified as a serum albumin-binding glycoprotein secreted by endothelial cells [15]. SPARC is involved in a number of cellular functions, including modulating cellular attachment, decreasing cellular adhesion, and inducing proliferation [16-19]. Even though SPARC has been implicated in tumorigenesis, the specific SPARC-mediated mechanisms involved in cancer have not been definitively elucidated, likely due to the diversity of SPARC functions. SPARC expression patterns appear to correspond with different outcomes in different cancer types. In some tumor types (*e.g.*, breast, melanoma, and glioblastoma), SPARC expression levels correlate with more aggressive behavior, while the opposite is true in other types (*e.g.*, ovarian, colorectal, and pancreatic) [19,20]. Since SPARC binds albumin, it was hypothesized that tumoral SPARC may sequester albumin-conjugated molecules, such as nanoparticle albumin-encapsulated (NAB)-paclitaxel [20,21]. SPARC may then facilitate the accumulation of NAB-paclitaxel in the tumor and potentially increase its effectiveness. Indeed, SPARC expression has been shown to correlate with response to NAB-paclitaxel in head and neck cancer patients [21]. However, further investigations are necessary to determine whether NAB-paclitaxel exploits SPARC expression to target tumor cells.

The goal of this study is to explore the pattern of SPARC expression and determine its prognostic significance in STS.

## Materials and methods

### Tissue selection

The pathology archive at the University of Arizona Medical Center (UAMC) was queried for sarcoma tissue samples surgically resected from patients at UAMC between 2000 and 2007. After review, 27 formalin-fixed paraffin-embedded (FFPE) tissue blocks representing a range of sarcoma histologies were identified, as outlined in Table 1. The specimens were selected based on their availability in the archives and suitability for immunohistochemistry (IHC). No information on clinical outcome was used *a priori* to select cases. Clinical data was obtained thereafter, allowing estimation of survival from date of diagnosis of unresectable or metastatic disease to the date of either death or last date of follow-up.

**Table 1:** Frequency of SPARC expression by sarcoma subtype

**Subtype**	**# of Specimens**	**Low SPARC (%)**	**High SPARC (%)**
Liposarcoma	11	5 (45)	6 (55)
Leiomyosarcoma	5	3 (60)	2 (40)
Synovial sarcoma	4	2 (50)	2 (50)
Angiosarcoma	1	0 (0)	1 (100)
Rhabdomyosarcoma	1	0 (0)	1 (100)
Primitive Neuroectodermal Tumor (PNET/Ewing’s)	5	2 (40)	3 (60)
Total	27	12 (44)	15 (56)

### Immunohistochemistry, SPARC staining and scoring

The Tissue Acquisition and Cellular/Molecular Analysis Shared Service (TACMASS) core facility at the University of Arizona Cancer Center (UACC) cut and prepared 5-micron thick slides from each tissue block. IHC for SPARC was performed using mouse monoclonal antibody (Abnova, Taipei City, Taiwan). Human glioblastoma tissue was used as the positive control at a dilution of 1:300. Tissue sections were stained with Discovery XT Automated Immunostainer (Ventana Medical Systems, Inc., Tucson, AZ; VMSI) using VMSI-validated reagents for deparaffinization, cell conditioning (antigen retrieval with a borate-EDTA buffer), primary antibody staining, detection and amplification using a biotinylated-streptavidin-HRP and diaminobenzidine system and hematoxylin counterstaining. Following staining, slides were dehydrated through graded alcohols to xylene and coverslips were applied using mounting medium.

The reviewing pathologist was blinded with respect to any clinical information regarding the cases of interest. Staining of SPARC was reviewed and scored using pathology long scores [22,23]. The pathology long score is a semi-quantitative system that represents the percentage of positive stained tumor cells, ranging from 1 to 100%, that exhibit different staining intensities. The staining intensity scale ranges from 1+ to 3+, where 1+ represents low positivity; 2+ represents moderate positivity; and 3+ represents strong positivity. Long scores are calculated by multiplying the intensity by the percentage. As an example, if a specimen is scored as 1+ 80% and 2+ 20%, the long score would be 120 [(1 × 80) + (2 × 20)]. The maximum long score is 300, where 100% of the tumor specimen is exhibiting 3+ staining intensity. Patient specimens were classified as low-to-moderate SPARC (depicted as “Low SPARC”) or moderate-to-high SPARC (depicted as “High SPARC”) based on the following criterion: if at least 50% of the tumoral cells displayed 2+ staining (long score is between 150 and 300), the specimen was classified as High SPARC. Otherwise, the specimen was classified as Low SPARC (long score < 150).

### Patient survival information and statistical analysis

Patient medical records were reviewed to determine the date of diagnosis of unresectable or metastatic disease. The survival time was measured from date of diagnosis of unresectable or metastatic disease to date of death or date of last follow-up. SPARC expression levels were correlated with patients’ survival and results were represented using Kaplan-Meier survival analysis. Differences in survival of the two groups of patients were assessed using the log-rank test.

## Results

### SPARC expression levels do not correlate with specific STS histologies

The sarcoma tissue specimens were assessed for SPARC expression level via IHC. Approximately half of the specimens (56%; 15 out of 27) demonstrated “High SPARC” staining, as defined above; the remainder (44%; 12 out of 27) demonstrated “Low SPARC” staining (Table 1). In the set of specimens evaluated in this study, the level of SPARC expression did not correlate with the underlying STS histology of the tissue specimens; *i.e.* the level of SPARC expression did not segregate with respect to STS histology. Given the limited sample size, we cannot draw definitive conclusions in regards to association of SPARC and histology.

### Survival of sarcoma patients strongly segregate by SPARC expression level

**Figure 1:**
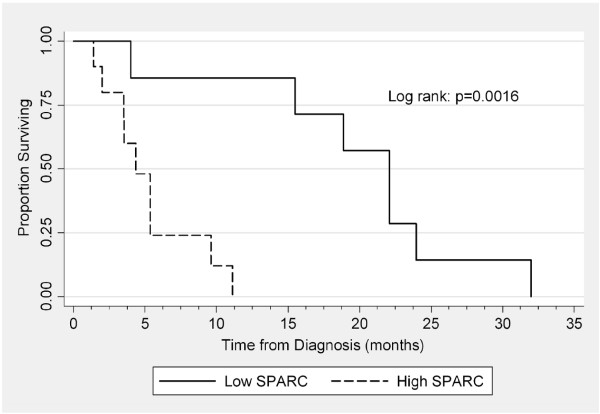
**Kaplan-Meier and log-rank analyses were used to compare survival of all patients segregated by SPARC expression level.** Median survival for the “Low SPARC” group is 22.1 months and “High SPARC” group is 4.4 months (Log rank: *p* = 0.0016).

To analyze the clinical significance of SPARC expression, we assessed the impact of SPARC expression levels on patient survival. Survival was defined as the total time from initial diagnosis of unresectable or metastatic disease to death or loss to follow-up. Data regarding date of diagnosis of unresectable or metastatic disease was determined for 17 out of 27 patients (data regarding date of diagnosis were unavailable for 10 patients). Only 6 of the 17 tumor samples were collected within 1 month of diagnosis of unresectable or metastatic disease; the remaining tumor samples were collected more than one month before or after the diagnosis of unresectable or metastatic disease. Kaplan-Meier and log-rank analyses were used to compare survival of patients (Figure 1). The median survival of patients in the Low SPARC group was 22.1 months (range 4-32 months), whereas the median survival of patients in the High SPARC group was 4.4 months (range 1.4-11.1 months). The difference in median survival between the two groups was statistically significant (*p* = 0.0016). These data are summarized in Table 2.

**Table 2:** Survival of patients by SPARC expression level

**Subtype**	**# of specimens (%)**	**Median survival (months)**	**Survival range (months)**
Low SPARC	12 (44)	22.1	4 – 32
High SPARC	15 (56)	4.4	1.4 – 11.1

## Discussion

We identified 27 tissue specimens representing a range of underlying STS histologies from the pathology archives at the University of Arizona Medical Center. We did not attempt to identify a specific subtype to study, but rather made a survey of STS specimens treated at our center. As would be expected, more common STS subtypes (liposarcoma, leiomyosarcoma, synovial sarcoma) made up the majority (20/27, 74%) of the specimens identified, with the remainder being less common STS subtypes. This is consistent with our intention of undertaking a preliminary survey of SPARC expression in STS.

About half of the specimens (56%) expressed moderate-to-high levels of SPARC. In our sample, SPARC expression did not clearly correlate with any specific STS histology, but we concede that the limited sample size prevents us from drawing definitive conclusions in this regard. What can be said, however, is that all histologies assessed demonstrated some specimens with elevated SPARC expression. Elevated SPARC expression is not peculiar to any particular subtype, but is observed in a variety of STS subtypes.

Tissue specimens were not collected at a specific timepoint in the clinical history of each patient. The relative timing of their collection was dictated by clinical considerations. This raises the question on whether elevated SPARC expression levels are involved in the etiology of STS (*i.e.*, occurs at disease baseline) or occur later as an epiphenomenon associated with disease progression. As noted above, eleven tumor specimens were collected more than one month before or after diagnosis of advanced disease. No obvious correlation of SPARC expression level with the relative time of sample acquisition was evident (data not shown). Since our investigation here does not specifically address the role of SPARC in the natural history of STS, this could be the focus of future studies.

To determine the clinical significance of SPARC expression level, we compared the survival of patients who expressed moderate-to-high and low-to-moderate levels of SPARC. Those with moderate-to-high levels of SPARC expression demonstrate inferior survival as compared with low SPARC expressors. The degree of segregation by SPARC level is statistically significant (*p* = 0.0016). While confirmation of our results in larger numbers and more STS subtypes is necessary, our current results lead us to hypothesize that SPARC may serve as a reliable prognostic factor in STS.

SPARC expression in STS may also be able to serve as a therapeutic target. Agents targeting SPARC-expressing tumors, such as NAB-paclitaxel (Abraxane, Celgene Corporation), have been designed to improve the therapeutic index of paclitaxel [24]. NAB-paclitaxel may act as a “Trojan horse,” with the albumin encapsulation serving to direct the paclitaxel chemotherapeutic agent to tumor cells via binding to SPARC. While taxane monotherapy has been shown to be active primarily in angiosarcoma [25,26], several reports have demonstrated that taxane combination therapy is active in STS (reviewed in [7]). Notably, the combination of docetaxel and gemcitabine has demonstrated superiority over gemcitabine monotherapy in a randomized phase 2 trial in STS [27]. As such, the combination of docetaxel and gemcitabine is widely adopted in STS management. While NAB-paclitaxel monotherapy has yielded somewhat discouraging results in STS [28], we believe that NAB-paclitaxel has not been adequately assessed in STS yet. Should the use of NAB-paclitaxel be further explored in STS, whether alone or in combination therapy, our current findings suggest that stratification by the extent of SPARC expression could be important in determining trial outcome. The marked differences in survival between low- and high-SPARC-expressing STS could mask beneficial anti-neoplastic effects, if the benefit is limited to one group. We suggest that the group with high-SPARC expression would be most likely to benefit from such an agent.

## Conclusions

Our investigations highlight several important findings: i) a significant proportion of the STS specimens investigated exhibit elevated SPARC expression; ii) elevated SPARC did not correlate with a specific underlying STS histology; iii) survival of STS patients segregated strongly by SPARC expression; and iv) SPARC levels are inversely associated with survival of patients with STS. Given our small sample size, however, further studies should be directed at confirming these findings in a larger number and variety of STS subtypes, as well as exploring the temporal course of SPARC expression in the natural history of STS.

## Abbreviations

FFPE: Formalin-fixed paraffin-embedded; GIST: Gastrointestinal stromal tumor; IHC: Immunohistochemistry; NAB-paclitaxel: Nanoparticle albumin-encapsulated paclitaxel; SPARC: Serum protein acidic and rich in cysteine; STS: Soft-tissue sarcoma; UACC: University of Arizona Cancer Center; UAMC: University of Arizona Medical Center; VMSI: Ventana Medical Systems, Inc.

## Competing interests

The authors declare that they have no competing interests.

## Authors’ contributions

SSM and LDC conceived the study design, performed statistical analysis, and drafted the manuscript. RBN performed pathologic review and immunohistochemical analysis. All authors read and approved the final manuscript.
